# Year-Long Monitoring of Physico-Chemical and Biological Variables Provide a Comparative Baseline of Coral Reef Functioning in the Central Red Sea

**DOI:** 10.1371/journal.pone.0163939

**Published:** 2016-11-09

**Authors:** Anna Roik, Till Röthig, Cornelia Roder, Maren Ziegler, Stephan G. Kremb, Christian R. Voolstra

**Affiliations:** Red Sea Research Center, Division of Biological and Environmental Science and Engineering, King Abdullah University of Science and Technology, Thuwal, 23955–6900, Saudi Arabia; Centro de Investigacion Cientifica y de Educacion Superior de Ensenada Division de Fisica Aplicada, MEXICO

## Abstract

Coral reefs in the central Red Sea are sparsely studied and *in situ* data on physico-chemical and key biotic variables that provide an important comparative baseline are missing. To address this gap, we simultaneously monitored three reefs along a cross-shelf gradient for an entire year over four seasons, collecting data on currents, temperature, salinity, dissolved oxygen (DO), chlorophyll-a, turbidity, inorganic nutrients, sedimentation, bacterial communities of reef water, and bacterial and algal composition of epilithic biofilms. Summer temperature (29–33°C) and salinity (39 PSU) exceeded average global maxima for coral reefs, whereas DO concentration was low (2–4 mg L^-1^). While temperature and salinity differences were most pronounced between seasons, DO, chlorophyll-a, turbidity, and sedimentation varied most between reefs. Similarly, biotic communities were highly dynamic between reefs and seasons. Differences in bacterial biofilms were driven by four abundant families: Rhodobacteraceae, Flavobacteriaceae, Flammeovirgaceae, and Pseudanabaenaceae. In algal biofilms, green crusts, brown crusts, and crustose coralline algae were most abundant and accounted for most of the variability of the communities. Higher bacterial diversity of biofilms coincided with increased algal cover during spring and summer. By employing multivariate matching, we identified temperature, salinity, DO, and chlorophyll-a as the main contributing physico-chemical drivers of biotic community structures. These parameters are forecast to change most with the progression of ocean warming and increased nutrient input, which suggests an effect on the recruitment of Red Sea benthic communities as a result of climate change and anthropogenic influence. In conclusion, our study provides insight into coral reef functioning in the Red Sea and a comparative baseline to support coral reef studies in the region.

## Introduction

Shallow-water coral reefs are marine ecosystems of high biodiversity and high economic value [1]. They depend on symbiotic reef-building corals that critically rely on sunlight, and are limited to the warm and oligotrophic conditions of equatorial oceans. Coral reefs exist in regions where seasonality, upwelling, or internal waves drive the variability of critical physico-chemical variables such as temperature, salinity, dissolved oxygen, and nutrient supply [2–4]. Distances of reefs to the shore are often associated with differences in certain physico-chemical properties; e.g., gradients of nutrient concentrations, sedimentation, and turbidity are common across spatial scales [5–7]. Physical forces such as hydrodynamics can alter physico-chemical variables by driving fluxes, the residence time of sea water in reef systems, and the exchange of coastal reef water with the open sea [8,9]. As a consequence, physico-chemical variables of coral reef ecosystems can fluctuate from favorable to less favorable conditions on spatial scales of kilometers and on temporal scales of months with seasonality [10,11].

Coral reefs are considered to be among the most sensitive ecosystems in regard to changing environmental conditions [12]. Many studies have focused on how anomalies and changes of physico-chemical variables (such as above-average summer temperatures or increased coastal nutrient input) can drive shifts in the ecology and composition of coral reef benthic invertebrate assemblages and associated reef fish communities [13–17]. The fundamental role of bacterial communities in coral reefs is well recognized [18–20]. Many studies focus on the role of bacterial consortia associated with coral or sponge host organisms in symbiosis or disease [21–26]. However, less is known about the drivers and dynamics in microscopic assemblages of epilithic biofilms, such as epilithic bacterial communities (hereafter ‘bacterial biofilms’). These bacterial biofilms are ubiquitous on surfaces in coral reefs, contribute to productivity, biogeochemical cycles [27], and facilitate larval recruitment of key reef-organisms, such as reef-building corals [28–30]. Consequently, intact bacterial biofilms are important for maintaining coral reef functioning, but local anthropogenic stressors, such as terrestrial runoff [31] and eutrophication [32], and factors related to global climate change (e.g., rising temperature and declining pH) can induce changes in coral reef biofilm communities [33–35].

The algal component of biofilms (hereafter ‘algal biofilms’) is another crucial part of the coral reef benthos. Epilithic algal turfs and crusts comprise a great part of reef primary production and constitute an essential food source for grazing reef fish [36,37]. Epilithic algae provide substrate for bacterial growth with different algal exudates selecting for specific bacterial communities [38,39], which also impact coral recruitment. While algal turfs reduce the settlement of marine invertebrates and inhibit the survival of coral recruits [40–42], crusts of coralline algae promote and induce the settlement of coral larvae [43]. At a later succession stage certain algal taxa typically compete with corals for space [44]. Under unfavorable conditions (e.g., high nutrient concentrations or overfishing) this can result in a phase shift from a coral-dominated towards an algal-dominated reef community, entailing the degradation of reef habitat [45]. Similar to bacterial biofilms, algal biofilm communities are highly responsive to changes in the environment, e.g. to temperature variation or pollution [46–48].

Due to the geographic location of Red Sea, locked in between two arid landmasses, precipitation and riverine input are rare and temperature and salinity are high [49,50]. This creates a unique environmental setting for tropical coral reefs, but despite these challenging conditions coral reefs are abundant along the Red Sea coastlines. Reefs in the central Red Sea are exposed to physico-chemical changes driven by a seasonal cycle [49], as exemplified by the prominent variability of sea water temperatures [51]. These coral reefs commonly stretch over coastal platforms and form offshore reef structures and lagoonal inshore areas that give rise to spatial environmental gradients [52–54]. This setting offers the opportunity to explore spatio-temporal coral reef dynamics under unique conditions. As in other regions, coral reef studies in the Red Sea have typically targeted benthic assemblages, such as reef-building coral and fish communities [15,55–58]. Conversely, little is known about the composition and dynamics of microscopic biota such as bacterial and algal biofilms. However, first data on coral associated bacteria show variable microbiomes in response to natural environmental gradients [59] and anthropogenic stressors [60–63], indicating variability on the level of microscopic biota in Red Sea reefs.

In recent years, consequences of global climate change have been reported to affect coral reefs in the Red Sea. For instance, the Red Sea is already experiencing measurable ocean warming [64], is susceptible to coral bleaching [15], and there are indications for a temperature-related decrease in coral growth [54,65]. However, most data on physico-chemical conditions are still based on remote sensing or occasional sampling events [65–68], rather than on continuous and more accurate *in situ* measurements. With increasing local and global anthropogenic stressors, comprehensive studies are needed that simultaneously record multiple physical, chemical, and biotic variables *in situ* to disentangle spatio-temporal dynamics and to provide a baseline against which impacts can be measured [69–71].

The lack of *in situ* baseline data was an important motivation for monitoring and collecting continuous physico-chemical and biotic data in this study. We use these data to characterize the natural baselines of central Red Sea reef environments. We then link physico-chemical and biotic parameters to extract putative physico-chemical drivers that contribute insights into coral reef functioning. As such we address the natural environmental variability of coral reefs in the central Red Sea to provide a foundation for coral reef studies, which will aim at better understanding coral reef functioning in unique environments and estimating the impact of global and local environmental pressures in this region.

## Material and Methods

### Study sites and design

**Fig 1:**
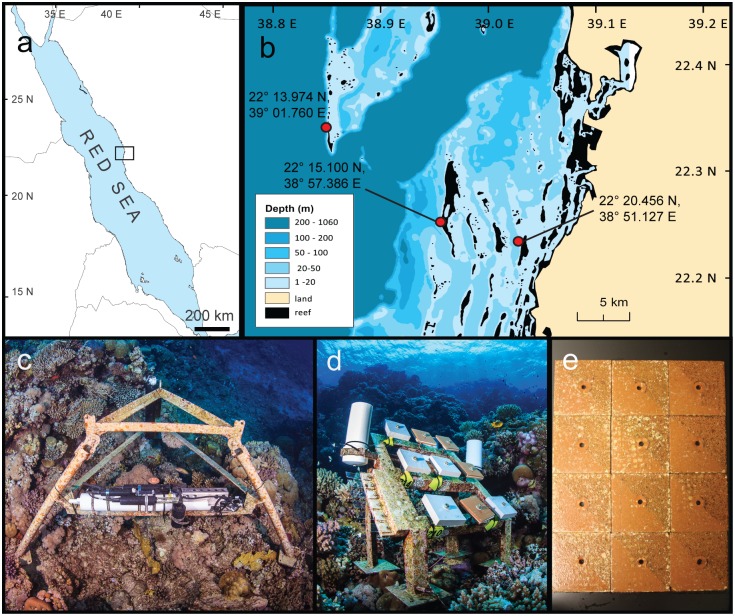
Map of study area and set up of coral reef monitoring sites in the central Red Sea. (a) The study area is located in the central Red Sea, (b) with three study sites (red markers) across the shelf. (c) Loggers (CTDs and ADCPs) for continuous data collection were moored to aluminum tripods fixed to the reef at 7–9 m depth. (d) Sediment traps and tiles were mounted on an aluminum frame fixed to the reef. (e) Sampled tiles after scraping off half of the biofilm for 16S rRNA gene amplification. Image credits: (a, b) Maha Khalil; (c, d) Tane Sinclair Taylor; (e) Stephan G. Kremb.

This study was conducted in the Saudi Arabian central Red Sea encompassing three reefs along a cross-shelf gradient using a balanced two-factor design (3 reefs and 4 seasons). Monitoring stations were set up at 7.5–9 m depth on the forereef slope of three geographically and visually distinct reef sites facing the open sea at 3 km (nearshore), 10 km (midshore), and 25 km (offshore) from the coast (Fig 1a and 1b). The nearshore site is surrounded by relatively turbid inshore waters and by other nearshore reefs in close proximity. The mid- and offshore sites are in well-mixed habitats that are fully exposed to the open sea. North-west winds are characteristic for the study area throughout the year [72]. Benthic cover differs between the three reefs with 24% live benthos in the nearshore site and ~70% in the midshore and offshore site. Calcifiers (corals and coralline algal crusts) are increasing in abundance from nearshore to offshore, while macro and turf algae are decreasing. Further descriptions of the reef habitats are available in a recent study detailing reef calcification by Roik *et al*. [54].

Each reef monitoring setup comprised one aluminum tripod for mooring of oceanographic instrumentation and one aluminum frame for deployment of sediment traps and terracotta tiles (Fig 1c and 1d). Four seasons were measured over intervals of 3 consecutive months from September 2012 to September 2013. Seasons were based on annual water temperature profiles from the region [51] as follows: fall starting on 15 September 2012, winter on 15 December 2012, spring on 15 March 2013, and summer on 15 June 2013. Given that the focus of this study is on providing environmental characteristics for each season, we decided that for ease of reference, seasons are best arranged from spring to winter. Discrete seasonal samples such as sediment traps and settlement tiles were recovered at the end of each season (± 5 d) (Table 1, S1 Table). The Saudi Coastguard Authority under the auspices of KAUST issued sailing permits to the sites that included sample collection.

**Table 1:** Overview of monitoring data from three reefs along a cross-shelf gradient over four seasons in the central Red Sea during 2012–2013.

Type of sampling	Variable	SPRING 2013			SUMMER 2013			FALL 2012			WINTER 2012–13		
		Near shore	Mid shore	Off shore	Near shore	Mid shore	Off shore	Near shore	Mid shore	Off shore	Near shore	Mid shore	Off shore
**ADCPs**	Current speed and direction	Deployment: March–June, Logging Frq: 10 min				Deployment: June–September, Logging Frq: 10 min		Deployment: September–December, Logging Frq: 10 min			Deployment:December–March, Logging Frq: 10 min		
**CTDs**	Temperature Salinity DO Turbidity Chlorophyll-a	Logging Frq: 60 min				Logging Frq: 60 min		Logging Frq: 60 min			Logging Frq: 60 min		

											ND		
											
											
**Reef water samples**	Phosphate Silicate Nitrate &Nitrite Ammonia				Sampling: September (2012), 6 Rep						Sampling: February (2012), 6 Rep		
			ND						ND				
					
					
	Reef water bacteria (total of 12 samples)	Sampling: June, 1 Rep			Sampling: September, 1 Rep			Sampling: December, 1 Rep			Sampling: March, 1 Rep		
**Sediment traps**	Sedi-mentation rate OC C:N ratio	Deployment: March—June, 3 Rep			Deployment: June—September, 3 Rep			Deployment: September—December, 1–3 Rep			Deployment: December—March, 3 Rep		
**Terracotta tiles**	Bacterial biofilm(total of 42 samples)	4 Rep			1–4 Rep			1–4 Rep			4 Rep		
	Algal biofilm(total of 48 samples)												

This overview shows deployment months and replicate numbers from continuous logging and discrete/seasonal sampling events. Continuous CTD and current data sets per reef and season contain 2000–2200 data points, apart from hatched areas, which contain 900–1500 data points. ADCP = Acoustic Doppler Current Profiler, CTD = Conductivity-temperature-depth recorder; DO = Dissolved oxygen; OC = organic content of sediments; C:N = carbon-nitrogen ratio of sediments; ND = no data; Frq = frequency; Rep = replicates

### Currents

Current speed (m s^-1^) and direction were measured continuously using Nortek AS Aquadopp Doppler current meters (Vangkronken, Norway). Instruments were moored vertically to tripod frames (Fig 1c) and exchanged every three months (Table 1). To reduce biofouling on the sensors a zinc oxide paste was applied (DESITIN^®^). Current profiles were recorded over a 0.5 m vertical profile (“bin”) at about 1.5 m above the substrate. Current speeds and directions were measured every 10 minutes. For each data point 20 measurements were averaged within 60 seconds, except in the midshore reef during spring measurements were taken every 5 minutes with 20 measurements averaged within 10 seconds. Rose plots were generated using the MATLAB (Release 2012b, The MathWorks, Inc., USA) function *wind_rose* [73] to show frequencies of current directions and speeds (m s^-1^) for each reef and season, and for the full year. Data are available from the Dryad Digital Repository: http://dx.doi.org/10.5061/dryad.9mj14.

### Temperature, salinity, dissolved oxygen, chlorophyll-a, and turbidity

Physico-chemical variables (temperature [°C], salinity [Practical Salinity Unit, PSU], dissolved oxygen [DO; mg L^-1^], turbidity [Nephelometric Turbidity Units, NTU], and chlorophyll-a [μg L^-1^]) were logged continuously over three-month intervals (Table 1) using conductivity-temperature-depth (CTD) recorders (SBE 16plusV2 SEACAT, RS-232, Seabird, USA, Fig 1c) equipped with a DO sensor (SBE 43, Seabird, USA), and an optical sensor for turbidity (700 nm) and chlorophyll-a fluorescence (ex/em: 470/695 nm; ECO FLNTU, WETlabs, USA). Sensors were fitted with automatic wipers counteracting biofouling on the sensor optics. Sampling frequency was set to 60-minutes intervals recording averages over 10 measurements. Continuous data from the CTDs were plotted as time series and as density plots using R function *geom_density* (kernel density estimation, default settings) [74].

### Dissolved inorganic nutrients

To report on phosphate, silicate, nitrate & nitrite, and ammonia we used samples from Ziegler et al. [75]. More specifically, discrete water samples were collected at each of the monitoring stations from 5 m and 10 m, once in winter (11 and 29 February 2012) and again at the end of summer (10 and 24 September 2012) and filtered over GF/F filters (0.7 μm; Whatman, USA).

### Sedimentation

Three replicate sediment traps (PVC tube traps of the dimensions: D = 8.2 cm, H = 22 cm) were placed 1 m above the substrate to measure sedimentation rates (Fig 1d). A funnel was fixed in the opening of the traps to reduce turbulence and minimize colonization by large marine organisms. Sediment traps were replaced every three months (Table 1); they were closed under water and transported to the lab on ice. Sea water including all sediment was filtered onto 0.22 μm PVDF filters (Millipore, Billerica, MA, USA) and filters were stored at -80°C until further processing. The samples were dried overnight at 40°C, weighed (Mettler Toledo, XS205, max 220 g, d = 0.01 g), and sedimentation rates were calculated as mg m^-2^ d^-1^. To assess the organic content (OC), sediments were ground using mortar and pestle. From each sediment trap a subsample was muffled at 550°C for 3 h, and the remaining ash-free dry weight was determined. Additional subsamples were used to measure organic carbon (C) and nitrogen (N) concentrations and isotopic signatures. Subsamples were acidified with 0.1 N HCl to remove inorganic C and CN content. Isotopic ratios (δ^13^C and δ^15^N) were analyzed relative to Pee Dee Belemnite standard and atmospheric nitrogen using an isotope ratio mass spectrometer (Delta plus XP, Thermo Finnigan, USA).

### Univariate analyses of physico-chemical variables

Univariate 2-factorial permutational MANOVAs (PERMANOVAs, Primer-E V6 [76]) were used to characterize the differences between the factors “reef” (3 levels: nearshore, midshore, and offshore) and “season” (4 levels: spring, summer, fall, and winter) for each of the 10 physico-chemical variables (current direction, current speed, temperature, salinity, DO, chlorophyll-a, turbidity, sedimentation rate, and OC and C:N ratios of sediments). Analyses were performed on monthly means of the continuous variables and based on Euclidian distances, type III partial sum of squares, 9,999 unrestricted permutations of raw data.

Inorganic nutrient species (phosphate, silicate, nitrate & nitrite, and ammonia) were analyzed in separate univariate 2-factorial ANOVAs testing the factors “reef” (3 levels: nearshore, midshore, and offshore) and “season” (2 levels: summer and winter), followed by Tukey’s HSD post-hoc tests where applicable (STATISTICA 10, StatSoft Inc. 2011).

### Bacterial communities of biofilm and reef water

To assess biofilms, terracotta tiles were first sanded on their non-glazed surface, autoclaved, and then deployed at the monitoring sites (surface dimensions: 10 x 10 cm, n = 4 tiles per site and season; Table 1). The tiles were attached to aluminum frames (Fig 1d) and aligned to the angle of the reef slope with the non-glazed side facing the water column. After recovery, tiles were rinsed with filtered sea water (0.22 μm), wrapped in aluminum foil, snap-frozen in liquid nitrogen on the boat, and stored at -80°C. One half of each tile was used to characterize bacterial communities and to determine algal cover of the biofilm (see below).

Reef water bacterial communities were assessed as follows: water samples were taken with cubitainers (4 L, n = 1) in direct proximity of each monitoring setup at the end of each season on the day of the tile recovery. Water samples were transported on ice in the dark to the lab. From each sample, 1 L was filtered over a 0.22 μm Durapore PVDF filter (Millipore, Billerica, MA, USA) and filters were frozen at -80°C until DNA extraction. Half of each water filter was cut into small stripes with sterile razorblades and transferred into a 2 ml vial. After adding 400 μl AP1 buffer of DNeasy plant kit (Qiagen, Hilden, Germany), the samples were incubated on a rotating wheel for 20 min and subsequently extracted following the manufacturer’s protocol.

To assess bacterial biofilms, samples were retrieved from terracotta tiles. Frozen tiles were placed on ice, unwrapped, and half of each tile was scratched off with a sterile razorblade (Fig 1e). Each biofilm sample was transferred into a 2 ml vial, vortexed, and about 100 mg transferred into a fresh vial. Next, 400 μl AP1 buffer (DNeasy plant kit, Qiagen) was added to each sample and DNA was extracted following the manufacturer’s protocol. After extraction, DNA concentrations for each sample were quantified on a NanoDrop 2000C spectrophotometer (Thermo Fisher Scientific, Waltham, MA, USA). We used the primers 341F (5'-TCGTCGGCAGCGTCAGATGTGTATAAGAGACAGCCTACGGGNGGCWGCAG-3') and 805R (5'-GTCTCGTGGGCTCGGAGATGTGTATAAGAGACAGGACTACHVGGGTATCTAATCC-3’) that target the 16S V3 and V4 regions [77]. The primers contained Illumina adapter overhangs used for subsequent indexing (underlined above; Illumina, San Diego, CA, USA). PCRs were performed in triplicate (with 5–14 ng DNA) using KAPA HiFi HotStart ReadyMix (KAPA Biosystems, Wilmington, MA, USA) with a final primer concentration of 0.2 μM and a total volume adjusted to 20 μl with RNase-free water. The amplification cycling temperatures were one cycle at 95°C for 3 min, 25 cycles each at 98°C for 30 sec, 55°C for 30 sec, and 72°C for 30 sec, followed by a final extension step at 72°C for 5 min. PCR products from one of the triplicates were visually assessed via 1% agarose gel electrophoresis with 10 μl per sample. Subsequently, triplicates were combined, PCR products were cleaned, indexed (8 cycles of indexing PCR using Nextera XT indexing adapters), and cleaned again following the Illumina 16S guidelines for MiSeq. All samples were quantified on the BioAnalyzer (Agilent Technologies, Santa Clara, CA, USA) and by Qubit (Quant-IT dsDNA Broad Range Assay Kit; Invitrogen, Carlsbad, CA, USA) and pooled in equimolar ratios. Sequencing was performed using the Illumina SBS technology for MiSeq at 8 pM and 10% phiX.

The software mothur (version 1.34.0, [78]) was used for 16S rRNA gene analysis. Sequence reads were split according to barcodes, contigs were built, singletons (n = 1 over all samples) were removed, a preclustering step (2 bp difference, [79]) was implemented, quality trimming was performed, and the data were aligned against SILVA (release 119, [80]). Chimeric sequences were removed using UCHIME as implemented in mothur [81], and unwanted sequences (chloroplasts, mitochondria, archaea, eukaryotes, unknown), classified against Greengenes [82] with a bootstrap of 60, were removed. Next, sequences of each sample were subsampled to 1,068 sequences, which eliminated six samples harboring from 7–207 sequences, resulting in 42 biofilm samples. Sequences determined in this study have been deposited in the NCBI Sequence Read Archive under accession number PRJNA306204 (http://www.ncbi.nlm.nih.gov/bioproject/PRJNA306204).

A 97% similarity cutoff was chosen to obtain Operational Taxonomic Units (OTUs) using the average neighbor algorithm in mothur. OTUs were classified based on their most abundant sequence. Stacked column plots were created based on the relative abundances of OTUs in the taxonomic families for each reef and season. To characterize and compare bacterial community composition in reef water and biofilms, mothur was used to derive the numbers of shared OTUs between biofilm and water samples via Venn diagrams. Mothur was further used to calculate Chao1 richness estimator [83] and the Inverse Simpson’s diversity index over each reef and season.

Due to different replication numbers for biofilm (n = 1 to 4) and water samples (n = 1), bacterial communities were evaluated using different statistical approaches. Alpha diversity indices of reef water communities were compared by two 1-way Kruskal-Wallis ANOVAs (first pooled for “reef”, second for “season”) followed by 2-tailed multiple comparisons tests where indicated. Two-factorial ANOVAs (for the factors “reef” and “season”) were performed for biofilms comparing alpha diversity indices. Where applicable, Tukey’s HSD post-hoc tests were conducted.

Reef water OTU community data were ln(x + 1) transformed and tested using two 1-factorial PERMANOVAs (one each for the factors “reef” and “season”). Test designs were based on Bray-Curtis similarities, partial sum of squares type III, 9,999 permutations of residuals under a reduced model using Monte-Carlo simulations, and followed by pair-wise tests where applicable. OTU based biofilm data were ln(x + 1) transformed and tested with a 2-factorial PERMANOVA for differences within each of the factors “reef” and “season”. Both data sets, bacterial communities of reef water and biofilms, were visualized in a non-Metric Multidimensional Scaling (nMDS) plot based on Bray-Curtis similarities. To test for differential abundance between “reefs” and “seasons”, the OTU data set was filtered to retain all OTUs present in > 50% of samples. This resulted in 72 reef water OTUs and 163 biofilm OTUs which were subjected to non-parametric Mack-Skillings analyses at a *p*-value cutoff of 0.01 (MeV V4.9, [84]).

### Algal biofilm communities

After each three-month deployment period, algal biofilms on recovered terracotta tiles were photographed with a stereomicroscope (Discovery.V20 SteREO and AxioCam MRm, Zeiss, Germany). To quantify the cover of functional categories overgrowing each tile, four to five randomly photographed subsections (1.4 x 1 cm) were examined using image-based analysis (CPCe software 4.1 [85]). In each subsample, the underlying organisms under 20 randomly selected points were assigned to one of nine functional categories: open space, filamentous algae, crustose coralline algae (CCA), green crusts (non-coralline light green crusts), red crusts (non-coralline red crusts), brown crusts (non-coralline dark-green and brownish crusts), cyanobacteria, red macroalgae (fleshy upright red algae), and sessile invertebrates [86]. Abundance counts for each tile were calculated as means of the randomly photographed subsections, converted to percent cover, and visualized in stack column plots per reef and season (n = 4).

Algae community data were statistically analyzed using the same test design as for bacterial biofilms. Additionally, a SIMilarity PERcentage (SIMPER) analysis (Primer-E V6, [87]) was conducted, and each of the nine algal categories was tested for differential abundance between “reefs” and “seasons” at a *p*-value cutoff of 0.05.

### Analyses of multivariate physico-chemical data and ‘biological-environmental’ matching

Analyses to characterize the overall physico-chemical conditions at the reef sites were based on 10 physico-chemical variables (current direction, current speed, temperature, salinity, DO, chlorophyll-a, turbidity, sedimentation rate, OC, and C:N ratio of sediments). First, significant correlations between physico-chemical variables were determined using Spearman's rank correlation analysis (STATISTICA 10, Stat Soft Inc. 2011). Second, environmental data were tested for differences between “reefs” and “seasons” with a 2-factorial PERMANOVA (based on square root transformed, normalized Euclidian distances, type III partial sum of squares, and 9,999 permutations of residuals under a reduced model) and visualized using an nMDS plot.

To link multivariate physico-chemical to biotic data (reef water bacteria, bacterial biofilm, and algal biofilm), biological-environmental (BIOENV) matching was performed in Primer-E V6 [87], based on Spearman’s rank correlations and 999 permutations. This routine was run three times to test the match between each of the resemblance matrices of biotic data with the distance matrix of physico-chemical data, and to determine those combinations of physico-chemical variables that best explained the structure in the biotic data.

## Results

We monitored three reefs along a cross-shelf gradient in the central Red Sea over an entire year and provide a detailed description of the seasonal dynamics of physico-chemical and biotic properties in the three reef habitats (Fig 1). We organize the data starting with physico-chemical variables and continuing with bi-annually measured inorganic nutrients and seasonal sedimentation. We then present data on bacterial community structure from the surrounding reef water. We further provide a first account of biotic communities, such as bacterial and algal biofilms. Our analyses explore the spatio-temporal dynamics of all physico-chemical and biotic variables and their interactions.

### Currents

**Fig 2:**
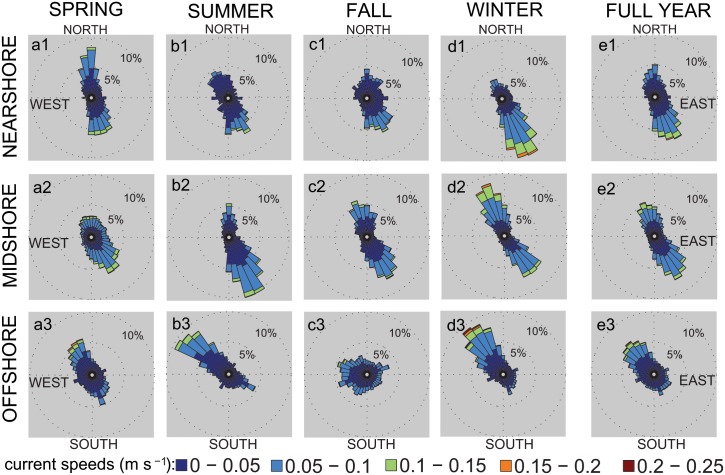
Current profiles from three coral reefs along a cross-shelf gradient over four seasons in the central Red Sea during 2012–2013. Rose plots display seasonal current profiles and a full year profile for each of the study sites (nearshore, midshore, offshore). Lengths of bars show the frequencies of current directions, angles indicate the direction where currents come from, and current speeds are coded by color. (Seasons: spring 2013, summer 2013, fall 2012, winter 2012–13)

We recorded current directions and speeds in proximity (1.5 m) to the reef. Across all reefs, the main axis of current direction was parallel to the shore (NW to SE). Over the year, the offshore reef was dominated by currents from NW to SE (Fig 2e3), whereas the flow in the nearshore reef was inverse with a main direction from SE to NW (Fig 2e1). Currents changing between both directions and of similar frequencies characterized the midshore reef (Fig 2e2). Current speeds reached maxima of 0.3 m s^-1^ at the offshore reef, which also experienced higher frequencies of strong currents compared to the other reef sites (Table 2). Current directions significantly differed between reefs, but not between seasons, and current speed significantly differed between reefs and seasons (Table 3). Over all reefs, current speeds mostly ranged between 0 and 0.1 m s^-1^ and significantly increased in winter compared with current speeds in fall (Table 3 and Fig 2).

**Table 2:** Summary of physico-chemical variables of three coral reefs along a cross-shelf gradient over four seasons in the central Red Sea during 2012–2013.

Variable	SPRING 2013			SUMMER 2013			FALL 2012			WINTER 2012–13		
	Near-shore	Mid-shore	Off-shore	Near-shore	Mid-shore	Off-shore	Near-shore	Mid-shore	Off-shore	Near-shore	Mid-shore	Off-shore
**Current speed [m s**^**-1**^**]**												
**Mean**	0.04	0.05	0.03	0.03	0.04	0.03	0.03	0.04	0.04	0.05	0.05	0.04
**SD**	0.03	0.03	0.03	0.02	0.03	0.02	0.02	0.03	0.02	0.03	0.03	0.03
**Max.**	0.16	0.22	0.22	0.14	0.17	0.17	0.18	0.22	0.19	0.19	0.17	0.30
**Temperature [°C]**												
**Mean**	28.22	28.10	27.81	31.93	30.82	30.78	30.19	30.30	30.39	26.14	26.53	26.52
**SD**	1.06	1.10	1.08	0.46	0.77	0.56	1.35	1.02	1.05	0.63	0.57	0.58
**Min.**	26.65	26.48	26.16	31.0	28.83	29.32	27.0	28.28	27.95	24.09	24.24	25.32
**Max.**	30.83	30.3	30.07	33.06	32.3	32.07	32.15	31.67	31.83	27.7	28.37	28.17
**Salinity [PSU]**												
**Mean**	39.2	38.99	38.94	39.50	39.25	39.30	39.59	39.52	39.57	39.30	39.33	39.10
**SD**	0.12	0.12	0.14	0.10	0.16	0.21	0.16	0.13	0.13	016	0.17	0.20
**Min.**	38.96	38.73	38.44	39.19	38.92	38.99	39.14	39.04	39.02	38.89	38.9	38.75
**Max.**	39.56	39.27	39.33	39.7	39.54	39.86	39.87	39.77	39.77	39.56	39.67	39.52
**Dissolved oxygen [mg L**^**-1**^**]**												
**Mean**	2.86	3.52	3.86	2.22	3.45	3.44	2.96	4.11	3.65	3.12	4.04	3.61
**SD**	0.51	0.67	0.59	0.77	0.63	0.55	0.76	1.04	0.59	0.32	0.46	0.83
**Min.**	1.14	0.7	2.29	0.09	0.45	1.51	1.63	1.95	1.92	2.81	2.41	0.07
**Max.**	4.2	4.66	5.01	3.86	4.79	4.69	6.11	8.87	5.22	4.05	5.3	5.24
**Turbidity [NTU]**												
**Mean**	0.41	0.49	0.41	0.49	0.35	0.63	0.47	0.48	0.44	0.53	0.47	0.20
**SD**	0.63	0.47	0.08	0.41	0.25	0.31	0.99	0.13	0.22	0.61	0.08	0.05
**Min.**	0	0	0.33	0.21	0.1	0.13	0.16	0	0.1	0.24	0.38	0.14
**Max.**	9.75	9.72	2.41	8.31	6.48	1.24	9.7	3.93	8.99	9.66	2.8	1.36
**Chlorophyll-a [μg L**^**-1**^**]**												
**Mean**	0.48	0.67	0.16	0.64	0.51	0.28	0.57	0.46	0.21	0.49	0.60	0.28
**SD**	0.16	0.11	0.07	0.19	0.26	0.11	0.27	0.13	0.09	0.23	0.14	0.10
**Min.**	0	0.31	0.07	0.3	0.16	0.03	0.12	0.11	0.06	0.09	0.24	0.11
**Max.**	2.03	3.4	0.65	2.67	1.53	0.75	3.18	1.86	0.69	3.4	1.32	0.93
**Inorganic nutrients [μM]**												
**Phosphate**												
**Mean**	-	-	-	0.05	0.05	0.06	-	-	-	0.09	0.09	0.09
**SD**	-	-	-	0.01	0.01	0.01	-	-	-	0.02	0.01	0.01
**Silicate**												
**Mean**	-	-	-	0.34	0.34	0.19	-	-	-	0.63	0.52	0.57
**SD**	-	-	-	0.08	0.07	0.03	-	-	-	0.12	0.06	0.09
**Nitrate/Nitrite**												
**Mean**	-	-	-	0.15	0.09	0.21	-	-	-	0.13	0.19	0.17
**SD**	-	-	-	0.08	0.07	0.13	-	-	-	0.03	0.03	0.08
**Ammonia**												
**Mean**	-	-	-	0.12	0.19	0.20	-	-	-	0.16	0.17	0.15
**SD**	-	-	-	0.16	0.1	0.1	-	-	-	0.12	0.08	0.11
**Sedimentation rate [mg m**^**-2**^ **day**^**-1**^**]**												
**Mean**	72.88	100.64	155.28	126.95	57.40	59.96	75.94	100.1	61.44	192.89	69.38	88.18
**SD**	45.9	42.35	18.84	45.60	14.59	21.13	11.05	-	13.50	20.30	8.43	19.44
**Organic content of sediments [mg m**^**-2**^ **day**^**-1**^**]**												
mean	12.84	14.33	18.02	18.88	9.59	12.47	13.18	15.56	6.01	28.51	10.30	10.79
**SD**	5.6	2.75	4.17	5.71	1.40	5.55	0.60	-	3.72	1.20	1.82	2.58
**Avg.. % of total sediments**	17.6	14.2	11.6	14.9	16.7	20.8	17.4	15.5	9.8	14.8	14.8	12.2
**C:N ratio of sediments**												
**Mean**	6.17	6.59	6.26	7.44	7.30	8.12	6.01	5.81	6.81	6.15	6.83	7.38
**SD**	0.39	0.63	1.26	0.33	0.53	0.22	0.12	-	0.55	0.17	0.59	1.48

Means, standard deviations (SD), minima and maxima (Min./Max.) summarize physico-chemical data

**Table 3:** Summary of univariate 2-factorial PERMANOVAs evaluating spatio-seasonal differences of physico-chemical variables in three coral reefs along a cross-shelf gradient in the central Red Sea during 2012–2013.

	Univariate 2-factorial PERMANOVAs					
	Reef		Season		Reef x Season	
	Pseudo-F	*p*	Pseudo-F	*p*	Pseudo-F	*p*
**Current direction**	**29.55**	**<0.001**	0.68	0.569	**6.75**	**<0.001**
**Current speed**	**10.07**	**0.001**	**7.97**	**0.001**	1.86	0.133
**Temperature**	0.40	0.675	**49.85**	**<0.001**	0.53	0.785
**Salinity**	**5.90**	**0.008**	**21.80**	**<0.001**	1.52	0.222
**Dissolved oxygen**	**21.09**	**<0.001**	**4.95**	**0.011**	1.30	0.297
**Turbidity**	0.66	0.544	0.38	0.793	1.84	0.116
**Chlorophyll-a**	**31.60**	**<0.001**	0.43	0.744	1.91	0.118
**Sedimentation rate**	**4.24**	**0.030**	**3.71**	**0.027**	**7.91**	**<0.001**
**OC**	**9.87**	**0.001**	2.10	0.128	**6.67**	**<0.001**
**C:N ratio**	2.85	0.081	**6.20**	**0.003**	0.65	0.689

OC = organic content of sediments, C:N = carbon/nitrogen ratio of sediments, significant variables (*p* < 0.05) in bold.

### CTD variables

**Fig 3:**
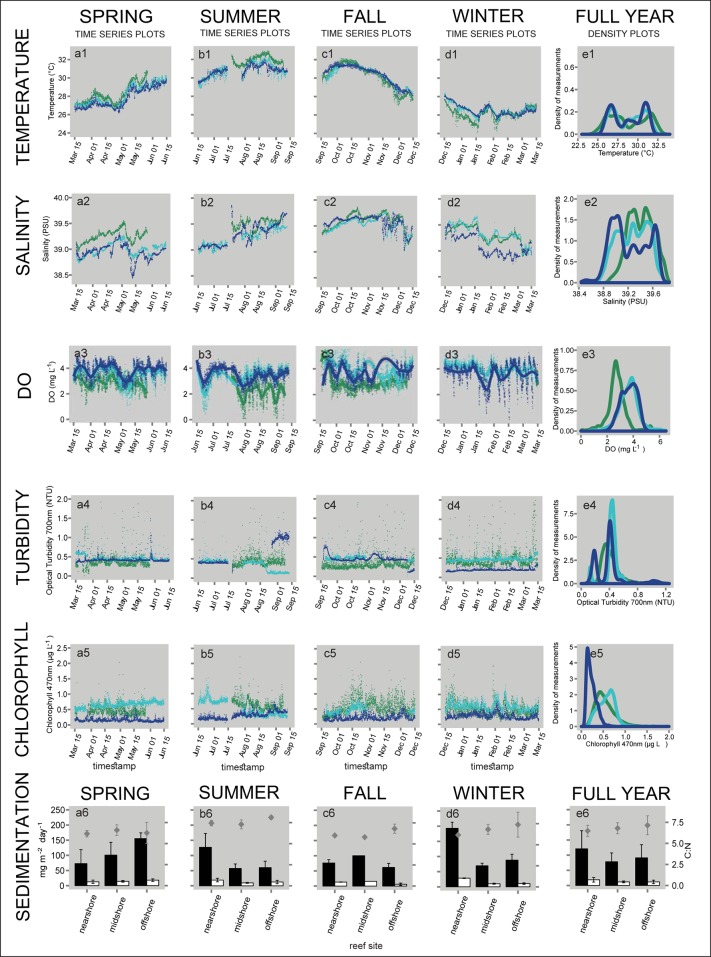
Physico-chemical variables of three coral reefs along a cross-shelf gradient over four seasons in the central Red Sea during 2012–2013. Continuously logged data of temperature (a1-d1), salinity (a2-d2), dissolved oxygen (DO; a3-d3), turbidity (a4-d4), and chlorophyll-a (a5–d5) are shown as time series plots over each season. The DO time series was fitted by polynomial regression (LOESS, span = 0.2). Plots in the last column (e1-e5) summarize full year data using density plots (kernel density estimation) to display frequency densities of data points observed in each reef. In the density plot of DO, the winter data set was excluded, as data from the nearshore reef were missing for almost the entire season. Sedimentation variables (a6-e6) are presented in bar plots (means ± SD). Green = nearshore reef; light blue = midshore reef; dark blue = offshore reef; black bars = sedimentation rate; white bars = organic content of sediments; diamonds = C:N ratio of sediments. (Seasons: spring 2013, summer 2013, fall 2012, winter 2012–13)

Physico-chemical variables were continuously logged in the three reefs along the cross-shelf gradient over the full year using CTDs. Data are displayed in time series and density plots (Fig 3). All seasonal means are summarized in Table 2. Differences in temperature and salinity were small between reefs and substantial over the year, whereas differences in DO, turbidity, and chlorophyll-a were pronounced between reefs, but not between seasons.

#### Temperature

Highest temperatures in summer reached 33°C and the lowest in winter reached 24°C (Table 2 and Fig 3a1–3d1). In spring and fall, temperatures transitioned and spanned a range of 8.97°C within three months. Density plots representing temperatures over the full year were bimodal (with two dominant frequency peaks) reflecting the substantial differences between summer and winter (Table 3 and Fig 3e1). Also, similarities and differences between sites were apparent in the plots: the temperature profile in the midshore reef was widely overlapping with the offshore site. At the same time nearshore temperatures were higher than in midshore and offshore during summer, and lower during winter (Fig 3b1 and 3d1).

#### Salinity

Salinity was lowest in spring (38.4 PSU) and increased over the summer reaching the highest values in fall (39.9 PSU; Table 2 and Fig 3a2 and 3c2). Most prominent seasonal differences were found in the offshore reef from 38.4 to 39.9 PSU (Fig 3e2). Salinity at the nearshore site was higher than at the other sites, except in fall when salinity values were very similar across all reefs (Fig 3c2). All site and seasonal differences were significant (Table 3), but variability was limited to a relatively small annual range of 1.4 PSU.

#### Dissolved oxygen (DO)

Seasonal means for DO included day and night measurements and ranged from 2.22 to 4.11 mg L^-1^ (Table 2). Seasonal differences were significant (Table 3). The variability over the full year was similar to the variability on shorter time scales: DO range for the full year was 8.81 mg L^-1^ and the range of DO within one reef and one season was up to 6.92 mg L^-1^. More obvious were the differences between sites, with lower DO at the nearshore reef compared to the midshore and offshore reefs (Table 3 and Fig 3a3–3e3).

#### Turbidity and chlorophyll-a

Turbidity values were lowest in winter (0.20 NTU) and highest in summer (0.63 NTU) at the offshore site. Chlorophyll-a values ranged between 0.16 μg L^-1^ and 0.67 μg L^-1^, recorded during spring in the offshore and midshore site, respectively (Table 2). Only site differences were statistically significant for chlorophyll-a (Table 3), characterized by a larger variability and higher values in the nearshore reef and lower values of decreasing variability with distance from shore (Fig 3a4–3e4 and 3a5–3e5).

### Dissolved inorganic nutrients

Bi-annual measurements of phosphate, silicate, nitrate & nitrite, and ammonia revealed overall low inorganic nutrients (Table 2). Nitrate & nitrite and ammonia did not vary significantly between reefs and seasons. The means for nitrate & nitrite were 0.16 μM and 0.17 μM for ammonia. Phosphate and silicate concentrations were significantly higher in winter (0.9 and 0.57 μM, respectively) than in summer (0.051 and 0.29 μM; both *p* < 0.001, ANOVA). Additionally, nearshore silicate was significantly higher compared to offshore (nearshore 0.48 μM, offshore 0.38 μM; *p* < 0.05, ANOVA).

### Sedimentation

Sediment traps were used to measure sedimentation rates, organic content (OC), and C:N ratios of the sediments (Fig 3a6–3e6). Rates differed significantly between reefs and seasons with a significant interaction between both factors (Table 3). The highest seasonal sedimentation rate was measured at the nearshore reef during winter (193 mg m^-2^ day^-1^), and the lowest at the midshore reef during summer (57 mg m^-2^ day^-1^; Table 2). During summer, fall, and winter, sedimentation rates decreased with distance from shore (Fig 3b6–3d6). Spring showed an inverse pattern with increasing sedimentation rate from nearshore to offshore (Fig 3a6).

OC content of sediments also significantly differed between reef sites, but not between seasons, and we found a significant interaction between both factors (Table 3). OC content ranged from 6.01 mg m^-2^ day^-1^ to 28.51 mg m^-2^ day^-1^ (Table 2), which resulted in an average contribution of 15.03% to total sediments. During spring and fall, the percentage of OC in sediments significantly decreased with increasing distance to shore (from 17.6 to 11.6%, and from 17.4 to 9.8% of total sediments, respectively; Table 2). This trend was reversed during summer: OC contribution was increasing with larger distance from shore (from 14.9 to 20.8%). During winter OC content was similar in all reefs (12.2–14.8%). The C:N ratio of sediments ranged between 6–8 across reefs and seasons (Table 2). C:N ratios in summer (7.3–8.1) were significantly higher compared to all other seasons (between 5.8 and 7.4, Tables 2 and 3). C:N ratios were similar between reefs, but increased with distance from shore (Fig 3a6–3e6).

### Community composition and dynamics of reef water bacteria and bacterial biofilms

MiSeq amplicon sequencing of the 16S rRNA gene from 12 reef water and 42 biofilm samples produced 4,183,963 sequences that clustered into 3,418 Operational Taxonomic Units (OTUs) at 97% (S2 Table). Good’s coverage [88] ranged between 0.92–0.97 for water samples and 0.73–0.95 for biofilm samples. The average number of OTUs per reef water and biofilm sample was 144 (± 15 SD) and 355 (± 58 SD), respectively. 408 OTUs were present in reef water, 2,929 in biofilms, and only an additional 81 OTUs (2%) were shared between them. Average diversity (Inverse Simpson’s index (ISI) 108.27) and richness (Chao1 670.93) were far higher in biofilm samples than in reef water samples (ISI 7.76; Chao1 220.99). Additional information on sequencing and OTU data of water and biofilm samples are presented in S3 Table and Table 4.

**Table 4:** Summary of reef water bacteria, bacterial biofilms, and algal biofilms from three coral reefs along a cross-shelf gradient in the central Red Sea over four seasons during 2012–2013.

Variable	SPRING 2013			SUMMER 2013			FALL 2012			WINTER 2012–13		
	Near-shore	Mid-shore	Off-shore	Near-shore	Mid-shore	Off-shore	Near-shore	Mid-shore	Off-shore	Near-shore	Mid-shore	Off-shore
**Reef water bacteria**												
**OTU**	119	139	125	121	132	122	117	132	125	99	104	154
**Chao1**	205	254	247	218	271	236	191	212	232	140	165	282
**ISI**	11	7	8	7	6	14	7	7	6	6	4	10
**Bacterial biofilms**												
**OTU**	415 (10)	377 (37)	346 (15)	274	446 (14)	363 (72)	319	293 (77)	311 (37)	347 (13)	316 (44)	365 (23)
**Chao1**	767 (61)	682 (91)	678 (70)	412	971 (63)	671 (228)	568	583 (273)	539 (170)	711 (72)	523 (152)	674 (80)
**ISI**	141 (21)	134 (35)	91(7)	71	164 (38)	119 (34)	99	63 (32)	87 (14)	89 (19)	99 (19)	108 (6)
**Algal biofilms**												
**SIMPER results (% contrib.)**	Open Space (29.1%)	Brown crusts (26.4%)	Brown crusts (27.6%)	Open Space (29.8%)	Brown crusts (26.4%)	Open Space (29.2%)	Open Space (11.0%)	Open Space (32.4%)	Open Space (27.5%)	Open Space (28.0%)	Open Space (32.3%)	Open Space (28.8%)
	Brown crusts (22.1%)	Open Space (22.1%)	Open Space (26.7%)	CCA (26.3%)	Open Space (25.7%)	Brown crusts (23.8)	CCA (24.5%)	Green crusts (21.5%)	CCA (26.1%)	Green crusts (20.3%)	Brown crusts (28.1%)	Green crusts (26.3%)
	CCA (21.7%)	Green crusts (17.7%)	Green crusts (18.4%)	Green crusts (21.2%)	Green crusts (20.5%)	CCA (20.8%)	Green crusts (16.9%)	Brown crusts (20.1%)	Green crusts (24.4%)	Brown crusts (19.4%)	Green crusts (13.5%)	Brown crusts (24.3%)
**Total % contrib.**	72.9%	66.2%	72.8%	77.2%	72.7%	73.8%	74.4%	74.1%	77.7%	67.8%	73.9%	79.2%

Biofilm and reef water bacterial communities are represented as mean (SD) number of OTUs, and alpha diversity indices: Chao1 richness estimator and Inverse Simpson’s diversity index (ISI). Values without SDs had only 1 replicate (see S1 Table). For algal biofilms, the three main contributors to similarity within each reef per season according to SIMilarity PERcentrage analysis (SIMPER) are listed; contrib. = contribution; CCA = crustose coralline algae.

#### Community composition and dynamics of reef water bacteria

**Fig 4:**
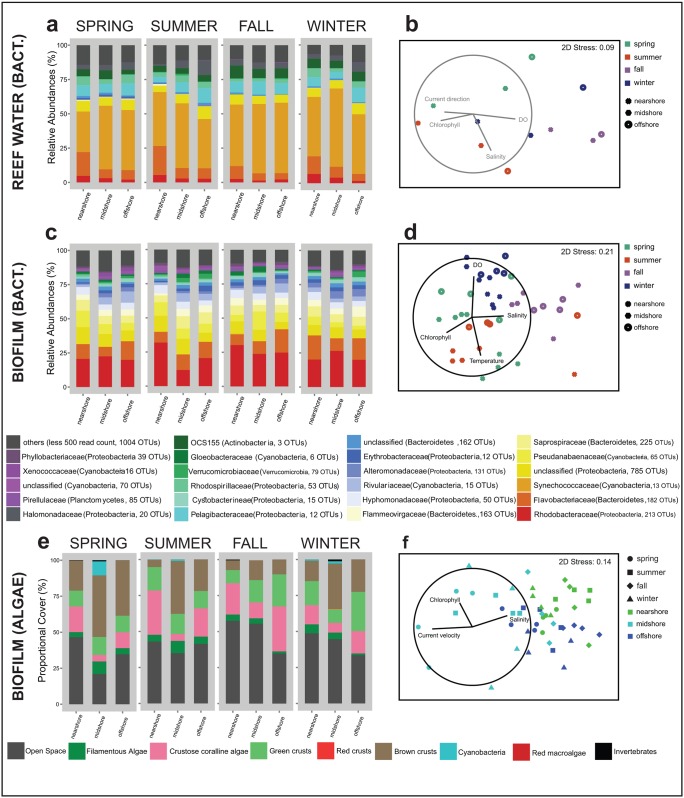
Community structure of reef water bacteria and bacterial and algal biofilms at three coral reefs along a cross-shelf gradient and over four seasons in the in the central Red Sea during 2012–2013. Stack column plots and non-Metric Multidimensional Scaling (nMDS) plots show the bacterial community composition of (a—b) reef water, and (c—d) biofilms and (e—f) algal composition of biofilms. Vectors (in b, d, f) represent combinations of physico-chemical variables, which best explain the structure of the biotic data (resulting from the Biological-environmental (BIOENV) matching routine). The length of the vectors indicates their correlation coefficients with the nMDS axes. Ordination of reef water bacteria (b) had no significant vectors. (Seasons: spring 2013, summer 2013, fall 2012, winter 2012–13)

Reef water bacterial communities were dominated by the family Synechococcaceae (Cyanobacteria; 30–55%). Together with Flavobacteriaceae (5–21%), Pelagibacteraceae (4–10%), OCS15 (3–9%), Halomonadaceae (1–10%), Rhodospirillaceae (1–6%), Rhodobacteraceae (1–7%), and unclassified Proteobacteria (4–9%), these bacterial families comprised up to 85% of the entire community (Fig 4a). Alpha diversity indices (Table 4) of reef water bacterial communities were not statistically different between reefs or seasons (*p* > 0.05, Kruskal-Wallis ANOVAs), but comparisons of reef water bacterial communities based on OTU abundance showed significant differences between seasons (Table 5, and Fig 4b). We were not able to identify any significant differences in abundances of OTUs on a spatial or seasonal scale, likely due to the absence of replicates for seawater bacterial communities.

**Table 5:** Summary of multivariate PERMANOVAs evaluating the spatio-seasonal structuring of biotic and physico-chemical data from three coral reefs along a cross-shelf gradient in the central Red Sea during 2012–2013.

	Reef		Season		Reef x Season	
	Pseudo-F	*p*	Pseudo-F	*p*	Pseudo-F	*p*
**Reef water bacterial communities**	1.40	0.176	2.04	**0.023**	-	-
**Bacterial biofilm communities**	3.27	**<0.001**	3.05	**<0.001**	1.57	**<0.001**
**Algal biofilm communities**	18.64	**<0.001**	8.54	**<0.001**	2.74	**0.001**
**Physico-chemical variables**	10.96	**<0.001**	7.34	**<0.001**	3.07	**<0.001**

Bacterial reef water communities were tested using 1-factorial PERMANOVAs with Monte Carlo simulations. Bacterial biofilms, algal biofilms, and 10 physico-chemical variables were tested with 2-factorial PERMANOVAs.

#### Community composition and dynamics of bacterial biofilms

Dominant bacterial families were Rhodobacteraceae (11–31%), Flavobacteriaceae (7–17%), unclassified Proteobacteria (5–12%), and Pseudanabaenaceae (3–12%), which together comprised about 50% of the biofilm communities (Fig 4c). Further, the bacterial families Saprospiraceae, Flammeovirgaceae, Hyphomonadaceae, Rivulariaceae, Alteromonadaceae, Erythrobacteraceae (each contributing 1–9%), unclassified Bacteroidetes, Cystobacterineae, Gloeobacteraceae, Verrucomicrobiaceae, Pirellulaceae, and unclassified Cyanobacteria (each with < 3%) added up to 85% of community composition (Fig 4c).

OTU-based alpha diversity of bacterial biofilm communities differed between seasons, with significantly lower ISI values in fall and winter and higher values in spring and summer (*p* < 0.05, ANOVA; Table 4). Bacterial community composition of biofilms significantly varied between reefs and seasons including a significant interaction (Table 5, Fig 4d). Pair-wise tests showed that differences were significant between almost all pairs of reefs and seasons (Table 5).

**Fig 5:**
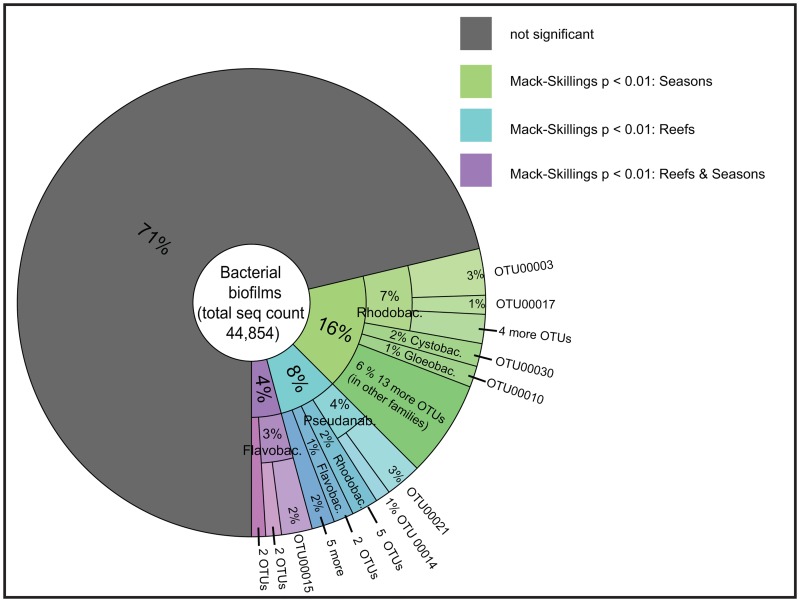
Proportions of differentially abundant bacterial OTUs between reefs and seasons. (Rhodobac. = Rhodobacteraceae; Cystobac. = Cystobacterineae; Gloeobac. = Gloeobacteraceae; Pseudanab. = Pseudanabaenaceae; Flavobac. = Flavobacteriaceae).

Overall, 19 OTUs differed in abundance between reefs, while 30 OTUs differed between seasons, and 5 OTUs differed significantly in both factors: between reefs and seasons (Table 6 and S4 Table). Sequence counts for significantly differential OTUs comprised 29% of all biofilm sequences (Fig 5) and displayed the following abundance patterns (S4 Table and Table 6): The most abundant OTUs that were significantly different between seasons belonged to the families Rhodobacteraceae (OTU00003: *Loktanella* sp.; unclassified OTU00017 and OTU00019), Cystobacterineae (unclassified OTU00030), and Gloeobacteraceae (OTU00010: *Gloeobacter* sp.). The overall most abundant OTU that significantly varyied between reefs was classified as *Halomicronema* sp. (OTU00021) in the family Pseudanabaenaceae. *Muricauda* sp. (OTU00015), a Flavobacteriaceae, varied between reefs and seasons. Bacterial taxa that significantly changed in abundance between the warmer and colder seasons were *Rhodovulum* sp. (OTU 00122), *Rhodobaca* sp. (OTU 00135), *Gloeobacter* sp. (OTU 00010), one unclassified bacterium of Flammeovirgaceae (OTU 00161), a bacterium of the order of Myxococcales (OTU 00232), and one from the family A4b (OTU 00419). All differentially abundant OTUs in reef biofilms were highly similar (94–99% identity) to sequences that had previously been encountered in marine and hypersaline environments (S4 Table; BLAST results).

**Table 6:** Bacterial OTUs and algal groups of biofilms that differed in abundance between reefs and seasons.

**Bacterial family (OTU)**	**Sequence counts per OTU**	***p* (reef)**	***p* (season)**	**Spatial pattern**	**Seasonal pattern**
*Abundance pattern*: *spatial gradient*					
Flavobacteriaceae(OTU00067)	301	< 0.01	0.03	increasing from NEARSHORE to OFFSHORE	-
Xenococcaceae(OTU 00048)	286	< 0.01	< 0.01		
Rhodobacteraceae(OTU 00097)	267	< 0.01	0.14		
Flavobacteriaceae(OTU 00134)	210	< 0.01	< 0.01		
Verrucomicrobiaceae(OTU 00206)	173	< 0.01	0.02		
Rhodobacteraceae(OTU 00145)	151	< 0.01	< 0.01		
Rhodobacteraceae(OTU 00331)	145	< 0.01	< 0.01		
Erythrobacteraceae(OTU 00058)	61	< 0.01	0.60		
Pseudanabaenaceae(OTU 00021)	1123	< 0.01	< 0.01	decreasing from NEARSHORE to OFFSHORE	
Flavobacteriaceae(OTU 00015)	951	< 0.01	0.00		
Flavobacteriaceae(OTU 00062)	317	< 0.01	0.05		
Rhodobacteraceae(OTU 00074)	232	< 0.01	0.04		
Hyphomonadaceae(OTU 00061)	181	< 0.01	0.34		
Rhodobacteraceae(OTU 00090)	131	< 0.01	0.40		
*Abundance pattern*: *other spatial*					
Pseudanabaenaceae(OTU 00014)	461	< 0.01	0.35	increased in MIDSHORE	-
unclassified Alphaproteobacteria(OTU 00065)	205	< 0.01	0.21		
Rhodobacteraceae(OTU 00259)	48	< 0.01	0.45		
Flavobacteriaceae(OTU 00031)	276	< 0.01	< 0.01	decreased in MIDSHORE	-
Flammeovirgaceae(OTU 00179)	88	< 0.01	0.04		
*Abundance pattern*: *increased during warmer seasons*					
Gloeobacteraceae(OTU 00010)	656	0.15	< 0.01	-	increased in SUMMER and FALL
Rhodobacteraceae(OTU 00122)	125	0.66	< 0.01		
unclassified Deltaproteobacteria (Myxococcales)(OTU 00232)	101	0.04	< 0.01		
Pseudanabaenaceae(OTU 00032)	78	0.94	< 0.01		
A4b(OTU 00419)	50	0.25	< 0.01		
Rhodobacteraceae(OTU 00019)	500	0.02	< 0.01	-	increased in FALL
Flammeovirgaceae(OTU 00161)	143	0.02	< 0.01		
Rhodobacteraceae(OTU 00135)	103	0.49	< 0.01		
*Abundance pattern*: *decreased during warmer seasons*					
Cystobacterineae(OTU 00030)	713	0.04	< 0.01	-	increased in SPRING and WINTER
Cohaesibacteraceae(OTU 00004)	412	0.21	< 0.01		
Xenococcaceae(OTU 00048)	286	< 0.01	< 0.01	-	increased in SPRING
Kiloniellaceae(OTU 00249)	214	< 0.01	< 0.01		
unclassified Alpharoteobacteria(OTU 00131)	170	0.20	< 0.01	-	increased in WINTER
Rhodobacteraceae(OTU 00145)	151	0.01	< 0.01		
Flammeovirgaceae(OTU 00155)	86	0.41	< 0.01		
Flavobacteriaceae(OTU 00201)	70	0.75	< 0.01		
Flammeovirgaceae(OTU 00137)	69	0.02	< 0.01		
Phycisphaeraceae(OTU 00083)	61	0.96	< 0.01		
*Abundance pattern*: *other seasonal*					
Alteromonadaceae(OTU 00093)	287	0.02	< 0.01	-	increased in FALL and WINTER
Flavobacteriaceae(OTU 00031)	276	< 0.01	< 0.01		
*Abundance pattern*: *temporal succession*					
Rhodobacteraceae(OTU 00003)	1441	0.04	< 0.01	-	increasing from SPRING to WINTER
Flavobacteriaceae(OTU 00015)	951	< 0.01	< 0.01		
Rhodobacteraceae(OTU 00017)	585	0.28	< 0.01		
Hyphomonadaceae(OTU 00016)	498	0.10	< 0.01		
Rivulariaceae(OTU 00059)	353	0.09	< 0.01	-	decreasing from SPRING to WINTER
Flavobacteriaceae(OTU 00134)	210	< 0.01	< 0.01		
Rhodobacteraceae(OTU 00053)	172	0.19	< 0.01		
Trueperaceae(OTU 00091)	163	0.02	< 0.01		
Pseudanabaenaceae(OTU 00055)	119	0.29	< 0.01		
Pseudanabaenaceae(OTU 00147)	109	0.74	< 0.01		
**Algal group**	**Count per group**	***p* (reef)**	***p* (season)**	**Spatial pattern**	**Seasonal pattern**
*Abundance pattern*: *spatial*					
Green crusts	712	0.03	0.08	increased in OFFSHORE	-
Cyanobacteria	55	0.02	0.46	increased in MIDSHORE	-
Filamentous Algae	225	0.04	0.35	decreased in OFFSHORE	-
*Abundance pattern*: *spatial and temporal*					
CCA	751	< 0.01	< 0.01	decreased in MIDSHORE	increased in SUMMER and FALL
Brown crusts	1056	< 0.01	< 0.01	increased in MIDSHORE	increased in SPRING and WINTER
Open space	1985	< 0.01	0.02	decreasing from NEARSHORE to OFFSHORE	increased in FALL

All significant results (Mack-Skillings tests; *p* < 0.01 for bacterial OTUs and *p* < 0.05 for algal groups) are denoted with a description of spatial and/or seasonal abundance pattern.

### Community composition and dynamics of algal biofilms

We assessed the composition of algal biofilms based on a total of 4,797 counts from 48 terracotta tiles. After three months exposure in the reefs, epilithic algae on average covered > 50% of each tile. The lowest algal cover (40–55%) occurred nearshore and midshore during fall and winter, whereas the highest algal cover (79%) occurred in the midshore reef during spring. Dominant algal categories were green crusts (10–30%), brown crusts (5–40%), and CCA (5–30%). Filamentous algae were present at low proportions (1–9%). Other rare categories, such as cyanobacteria (< 2%) and sessile invertebrates (~ 1%) were only found on tiles in nearshore and midshore reefs. We did not encounter red macro algae. Red crusts were absent on most tiles, except for the midshore reef during winter where the cover was < 1% (Fig 4e).

Green crusts, brown crusts, CCA, and open space, each contributed about 20 to 30% to the similarity within each reef and season, cumulatively explaining about 65 to 80% of the variability in algal communities (SIMPER, Table 4). Algal biofilm communities from the midshore reef varied more between seasons (Bray-Curtis similarity 87.27, SIMPER; Fig 4f) than algal communities from the nearshore and offshore reefs (92.99 and 91.89, respectively, SIMPER). Algal community composition was significantly different between reefs and seasons, including a significant interaction (Table 5). The algal community of each reef in each season was significantly different from all others (pairwise comparisons, *p* < 0.05), except summer and winter in the nearshore reef, and spring and summer in the offshore reef (*p* > 0.05) (Table 6 and S4 Table). Algal cover significantly increased with distance to shore. Seasonal variability of algal biofilms was characterized by a higher occurrence of brown crusts in spring and winter, and of CCA in fall, which coincided with a decrease of total algal cover.

### Physico-chemical environment and drivers of biotic communities in coral reefs

**Fig 6:**
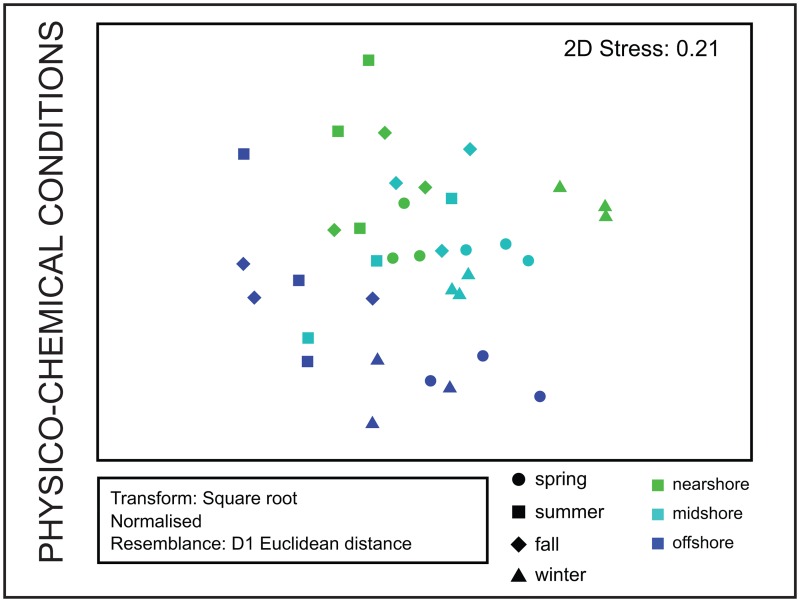
Structuring of reef habitats by physico-chemical conditions. Non-Metric Multidimensional Scaling plot illustrates the structure of reef environments based on 10 physico-chemical variables (current direction, current speed, temperature, salinity, dissolved oxygen, chlorophyll-a, turbidity, sedimentation rate, and organic content and C:N ratio of sediments).

Physico-chemical conditions structured reefs and seasons and were most different between summer and winter and the geographically most distant reefs, nearshore and offshore, as visualized in the nMDS plot (all *p* < 0.001; Table 5 and Fig 6). Several pairs of physico-chemical variables varied jointly or inversely between reefs and seasons (Table 7): Current speed and directions significantly correlated with chlorophyll-a. Current speed inversely correlated with temperature. Temperature significantly correlated with salinity and DO, while DO correlated with chlorophyll-a. Chlorophyll-a further correlated with turbidity and sedimentation rates with OC content of sediments.

**Table 7:** Correlations between physico-chemical variables and biological-environmental (BIOENV) matching.

**Physico-chemical variables (Spearman’s correlations)**					
**Variable 1**	**Variable 2**		**R**	**t(n-2)**	***p***
**Current speed**	Current directions		0.41	2.55	**0.016**
**Current speed**	Temperature		-0.51	-3.36	**0.002**
**Current speed**	Chlorophyll-a		0.34	2.05	**0.048**
**Current directions**	Chlorophyll-a		0.59	4.09	**<0.001**
**Temperature**	Salinity		0.50	3.34	**0.002**
**Temperature**	DO		-0.35	-2.07	**0.047**
**DO**	Chlorophyll-a		-0.40	-2.39	**0.023**
**Chlorophyll-a**	Turbidity		0.45	2.93	**0.006**
**Sedimentation rate**	OC		0.83	8.19	**<0.001**
**Biological-environmental correlations (BIOENV)**					
**Biotic similarity matrix**	**ρ**	***p***	**Correlated combination of variables**		
**Reef water bacterial communities**	0.37	0.196	Salinity, Chlorophyll-a, DO, Current direction		
**Biofilm bacterial communities**	0.47	**0.001**	Temperature, Salinity, Chlorophyll-a, DO		
**Biofilm algal communities**	0.35	**0.001**	Salinity, Chlorophyll-a, Current speed		

Significant results in **bold**; R = Spearman's correlation coefficient; t(n-2) = Spearman's rank correlation t-statistic; DO = dissolved oxygen; OC = organic content of sediments, ρ = Spearman’s correlation coefficient (BIOENV routine)

Differences in multivariate physico-chemical data correlated with differences in biotic communities. A combination of salinity, chlorophyll-a, DO, and current direction best explained variations in reef water bacterial communities, but this correlation was not significant (Table 7, Fig 4b). Matching physico-chemical data with bacterial biofilm communities resulted in a significant correlation, and the patterns were best explained by a combination of temperature, salinity, chlorophyll-a, and DO (Fig 4d). Differences in physico-chemical data and algal biofilm composition were best explained by the variables salinity, chlorophyll-a, and current speed that were also statistically significant (Fig 4f).

## Discussion

In this study we present a first account of physical, chemical, and biotic *in situ* data acquired simultaneously in coral reefs of the central Red Sea over the course of a full year. Our data revealed that the reefs in this region are exposed to a high degree of spatial (cross-shelf) and temporal (seasonal) variability. We uncovered connections between physico-chemical conditions and community structure of bacterial and algal biofilms, contributing valuable information on the potential major drivers of biotic coral reef processes in this naturally variable environment.

### Physico-chemical baseline data of coral reefs in the central Red Sea

Our *in situ* physico-chemical data show that coral reefs in the central Red Sea are subjected to summer temperature and salinity that exceed coral reef global average maxima [50] and to relatively low DO [89,90]. Turbidity and sedimentation rates were far below values reported from coral reefs elsewhere, especially from those that are frequently studied (e.g. Great Barrier Reef (GBR)), whereas chlorophyll-a and nutrients were similar to measurements from other coral reef regions [10,91]. Further, our data reveal a high degree of spatio-temporal variability: seasonality was primarily reflected in temperature and salinity, whereas DO, chlorophyll-a, and sedimentation varied over the spatial scale.

#### Currents

Derived from ocean model simulations, currents on the eastern coast of the central Red Sea are influenced by strong seasonal or permanent gyres and by the eastern boundary current that carries water masses from the south [92]. However, around reef platforms bathymetry and atmospheric forcing may be the strongest determinants for current properties [9,72]. Accordingly, the main current direction (NW to SE) at our offshore site was likely driven by north-west winds [72], while the reversed direction (SE to NW) in the nearshore reef may be related to the eastern boundary current (travelling northward) [92]. The currents around our study site are likely to transport nutrients and influence heat budgets, as indicated by the significant correlations of currents with chlorophyll-a and temperature. The offshore site receives water masses from the Red Sea basin, whereas water exchange between the nearshore reef and the basin may be limited. Elevated salinity in the nearshore reef supports this assumption, as it is likely caused by the longer residency time of water, resulting in higher relative evaporation rates. Water exchange between coral reefs and the open sea can play an important role in mediating stress events, such as rising salinity or excessive summer warming [93]. Hence, nearshore reefs in the central Red Sea may be at higher risk of experiencing episodes of environmental stress compared to the more distant reefs. Higher prevalence of bleached corals in nearshore than in offshore reefs during a coral bleaching event in 2010 and 2015 is consistent with this assumption [15,94].

#### Temperature and salinity

During summer we measured a highest seasonal mean temperature of 31.9°C and maxima of up to 33°C. The highest seasonal mean temperature exceeds the typical average maximum for coral reefs (29.5°C) [50] by 2.4°C and is already similar to conditions that are predicted for most other reefs worldwide by the end of this century [95]. Salinity at our study sites (38–39 PSU), while typical for the Red Sea [49], also exceeds global coral reef averages (34–35 PSU) [50,96].

Among all measured physico-chemical variables, temperature and salinity fluctuate most between seasons. For instance, the annual temperature range (9°C) is 2- to 4-fold higher than in most equatorial reefs (2–4°C in coral reefs in the Caribbean, Indo Pacific, and Pacific Ocean), and in a range with temperatures from more extreme regions that support coral habitats, such as the Sea of Oman (7°C) and the Persian/Arabian Gulf (12–20°C) [97]. Although salinity is high, its fluctuation is relatively low (range: 1.43 PSU) compared to tropical reefs that are influenced by riverine and precipitation input (e.g. salinity can vary by 5 to 10 PSU in a nearshore reef in the GBR [98]). In our study salinity might be driven by evaporation processes related to temperature, which could be concluded from the correlation of both variables, but also the possible influence of currents should be considered and deserves further investigation. The Red Sea is a semi-enclosed basin located between arid landmasses [49] that may be particularly affected by ocean warming, leading to even higher temperatures and salinity. Coral bleaching events are an indication that thermal limits of many coral species have already been reached [15]. The environmental data presented here will be an important contribution to quantify long-term effects of ocean warming in the central Red Sea.

#### Dissolved oxygen (DO)

DO concentrations in coral reefs are primarily driven by biological processes such as respiration and photosynthesis [89]. Lower DO in the nearshore reef suggests a predominance of heterotrophic organisms, such as sponges and other filter feeders, or heterotrophic bacteria, but also reduced water mixing close to shore. This study presents a DO range of ~1–6 mg L^-1^ which is derived from continuous data, including diurnal (elevated DO due to photosynthesis) and nocturnal values (lowered DO due to respiration). This is large in relation to the ranges from a majority of studies that only report on daytime measurements (6–9 mg L^-1^, e.g. [99,100]), but similar to day and night values measured in a high-latitude coral reef of Japan that span a similarly remarkable range from 1.3–11.1 mg L^-1^ [89]. Within the Red Sea averaged DO concentrations decrease from the north (6–7 mg L^-1^, offshore shallow waters [90]) to the central Red Sea (2.2–4.1 mg L^-1^, this study). This reduction in DO is likely driven by higher temperatures in the central Red Sea that decrease oxygen solubility.

Globally, DO concentrations are predicted to decrease and hypoxic environments to spread as a consequence of climate change [101]. Values of 2 mg L^-1^ DO and below have been characterized as hypoxic in the majority of studies, mostly for temperate regions [102]. As DO in the central Red Sea occasionally reaches such low concentrations, hypoxia may represent another challenge for Red Sea organisms in this region. However, given the lack of data and studies, it is not clear whether these low DO values are common in the central Red Sea or not.

#### Chlorophyll-a and dissolved inorganic nutrients

Chlorophyll-a concentration is frequently used as a proxy for primary production and nutrient availability in the water column [103]. Chlorophyll-a derived from remote sensing data shows that surface water concentrations in the Red Sea range from extreme oligotrophy (< 0.01 to 0.4 mg m^-3^) in the northern and northern-central Red Sea to chlorophyll-a concentrations exceeding typical coral reef conditions by an order of magnitude in the southern Red Sea (0.5–5.0 mg m^-3^) [104]. Accordingly, in the central Red Sea we found *in situ* chlorophyll-a and dissolved inorganic nutrients to be mostly in the range of values from other oligotrophic coral reef regions. Conditions at the nearshore and midshore reefs in our study area were similar to inshore reefs of the GBR (up to 0.7 μg L^-1^ over the full year; [10]), while concentrations in the offshore reef were lower (0.16 to 0.28 μg L^-1^) and in a range with more oligotrophic reef sites such as reef systems in Hawaii (up to 0.31 μg L^-1^) [105].

Low chlorophyll-a concentrations in our study area also reflect the limited availability of inorganic nutrients. Nitrogen species concentrations (nitrate & nitrite 0.16 μM; ammonia 0.17 μM) were comparably low (Hawaii, Phoenix islands, GBR, and Western Australia; 0.04–2.5 μM and 0.05–5.52 μM for nitrate & nitrite and ammonia, respectively [106]). Phosphate (0.07 μM) was among the lowest values reported for coral reefs (0.08–0.6 μM) [106].

#### Sedimentation and Turbidity

Sedimentation rates and turbidity were very low in the study area and decreased from nearshore to offshore following a common pattern of land-based sedimentation [6,7,107,108]. Turbidity is a proxy for suspended particulates in the water column that, depending on their organic content, are filtered or ingested by heterotrophic biota serving as a source of nutrition [109]. Because suspended particles inhibit light penetration, which impacts photosynthesis or smothers benthic organisms, high sedimentation loads are commonly regarded as stressors to coral reefs [110]. Sedimentation rates in Caribbean and Pacific coral reef habitats are considered ‘natural’ at 1–10 mg cm^-2^ day^-1^ [91], while stressful conditions start at around 70 mg cm^-2^ day^-1^ [111]. Sedimentation rates in the central Red Sea reefs are far below these values. Seasonal rates ranged between 0.0057–0.0193 mg cm^-2^ day^-1^, which is only ~ 2% of the lowest natural sedimentation rate recorded elsewhere [91]. Accordingly, seasonal averages of turbidity from the central Red Sea (0.20–0.63 NTU) are well below those from some sites in the GBR (0.6–7.0 NTU) [10].

Similar to chlorophyll-a, OC of sediments and turbidity showed no significant seasonal pattern that would indicate a period of higher productivity in the water column. However, the typical decrease of sedimentation rates from nearshore to offshore was reversed in spring. This may be related to the Indian Ocean monsoon, which causes dust storms and/or increases mixing in the water column during spring and fall [112]. Further monitoring is required to confirm if this pattern is reoccurring every year.

All C:N ratios of sediments were above the Redfield ratio (6.6) [113], which confirms that primary production in the central Red Sea is nitrogen limited [114]. This is also evident from low concentrations of nitrogen species in the study area. C:N ratios of particulates were even higher during summer compared to other seasons, indicating aggravated nitrogen limitation in this period [115].

### Biotic baseline data of coral reefs in the central Red Sea: reef water bacteria and bacterial and algal biofilms

We present a first account of basic biotic variables of coral reefs in the central Red Sea, including reef water bacteria, bacterial biofilms, and algal biofilms. The catalogue of bacterial taxa (S2 Table) and algal groups represents a first assessment of microscopic communities in naturally variable reef environments of the central Red Sea. Coral reef bacterial biofilms had a far higher species richness and diversity compared to Red Sea coral reef water or coral microbiomes [21,59,60,62]. Bacterial and algal biofilms were variable (29% of bacterial and 99% of algal communities significantly varied in abundance between reefs and seasons), and an increase in bacterial diversity during spring and summer coincided with significantly increased algal growth, supporting the notion of interaction between algal and bacterial communities via exudates [38,39]. Furthermore, significant variability between the warm and cool season provides insight into potential community changes associated with ocean warming. In the following the findings are discussed in detail.

#### Composition and dynamics of reef water bacteria

Reef water bacterial communities at our study sites were similar to those reported from other oceans [116,117]. Communities were dominated by the cyanobacterial family Synechococcaceae, which is characteristic for open sea surface water across the Red Sea [67,118]. Synechococcaceae are particularly adapted to oligotrophic environments and are a major primary producer in oligotrophic waters [119]. Similarly, Pelagibacteracaea, another abundant group in our samples, are associated with oligotrophic conditions [120]. Reef water bacterial community structure differed between seasons, but remained stable across reefs. This lack of spatial differences but strong seasonality may indicate minor land-based influences in our study area, given that reef water bacterial communities in areas of pollution are shown to change along spatial gradients and lack seasonal differences [121].

#### Composition and dynamics of bacterial biofilms

Epilithic bacterial biofilms in coral reefs have been characterized using molecular tools on spatial and temporal scales in Sulawesi, Indonesia [32] and in the GBR [28,31,35,122]. These studies focused on CCA associated bacteria or epilithic biofilm communities along a gradient of eutrophication or terrestrial runoff. To date, little is known about bacterial biofilm and reef water community structure and their responses to natural environmental fluctuations in little impacted environments.

We show that in the central Red Sea five bacterial phyla dominate biofilms over all reef sites and seasons. Of these, Proteobacteria, Bacteroidetes, and Cyanobacteria were previously described from coral reef biofilms in the GBR, and Verrucomicrobia were previously found in coral reef sediments [31,123] and in marine biofilms from temperate and polar regions [124,125]. The last bacterial phylum, Planctomycetes, was identified in estuarine biofilms [126] and on the surface of red algae [127]. On the family level, Rhodobacteraceae (Proteobacteria) and Flavobacteriaceae (Bacteroidetes) were most prevalent in Red Sea biofilms. Both families were found in coral reef biofilms before [28,35,128] and were associated with community shifts along a water quality gradient [31]. Rhodobacteraceae are known as rapid surface colonizers and are considered to be involved in the formation of marine biofilms [129]. They play diverse roles in benthic community structuring, with a few species enhancing coral recruitment [128], but other species being reported as pathogenic opportunists in coral disease [130–132].

Bacterial biofilm diversity in this study was at least 10-fold higher than bacterial diversity in reef water, and also in relation to reef water and coral microbiomes reported in other Red Sea studies [21,60,62]. Implications of this high bacterial diversity are still unknown and warrant further study of bacterial biofilms.

Similar to findings for reef water bacterial communities, studies showed that seasonality of biofilm communities was minor or not detectable along nutrient or pollution gradients in coral reef systems, while the spatial gradient was strong [31,32]. This is in contrast to bacterial biofilms in our study area that displayed high seasonality and low spatial dynamics. The prominent seasonal response may be interpreted as a natural pattern in a putatively less impacted reef area. This is corroborated by the observation that all differentially abundant OTUs were previously encountered in marine environments (see S4 Table) without any apparent link to anthropogenic sources [61]. Lastly, this study identified several bacterial OTUs that were significantly increased or decreased in the warmer seasons. These OTUs may be temperature sensitive and can presumably indicate community shifts caused by temperature changes.

#### Composition and dynamics of algal biofilms

Our biofilm data includes algal assemblages following a three months succession from a cross-shelf gradient over four seasons. Composition of algal biofilms plays an essential role in coral and other invertebrates settlement and the survival of recruits [40,43]. Brown or green algal crusts or turfs, which contributed up to 70% to algal communities in this study, are typically negatively associated with coral larval recruitment [133]. Some CCA have a beneficial effect on coral recruitment and survival [133], but these algae were less abundant in this study. CCA commonly dominate offshore environments, but in our data offshore and midshore environments showed similar amounts of CCA [134,135]. Our data represent algal settlement patterns on smooth and light exposed surfaces, where brown and green algae may have an advantage over CCA, which proliferate in low light environments [86,136]. Other algal groups such as red algae and red crusts were almost absent from the exposed settlement tiles, presumably because they also favor sheltered environments [86].

In our study, algal community composition significantly differed between reefs and seasons, confirming that algal communities and their biomass are highly dynamic [48,134,137]. Algal cover and bacterial diversity were highest during spring and summer coinciding with the timing of coral reproduction. This overlap with the coral spawning season in the central Red Sea (April to June) [138] indicates that algal community patterns may potentially influence the settlement behavior and success of coral larvae.

### Physico-chemical drivers of biotic communities in the central Red Sea

Increasing the understanding of environmental variability in coral reefs is essential to predicting ecosystem response to environmental change [12,139]. While addressing single physico-chemical variables in isolation may provide some insight, the analysis of cumulative effects from multiple variables is of relevance to gain a more complete understanding of complex ecological systems [140]. Our study provided an opportunity to match the simultaneously collected physico-chemical and biotic data and to explore interactions between physico-chemical conditions and the biotic realm *in situ*.

Although nutrient enrichment or pollution related factors were shown to be the only drivers of bacterial communities in coral reefs elsewhere [31,32,121,141], our results suggest that in less disturbed environmental settings, bacterial biofilms are influenced by a combination of temperature, salinity, DO, but also chlorophyll-a. *Ex situ* experiments confirmed a temperature induced regulation of bacterial biofilm composition and physiology [33,35,142]. Also, changes in salinity and DO were shown to influence biofilm and water column bacteria in estuaries [143–145].

The spatio-temporal structuring of algal biofilm communities at our sites were best explained by salinity, chlorophyll-a, and current speed. We expected chlorophyll-a to be associated with differences in algal composition, given that irradiance together with nutrient availability (both variables that are related to chlorophyll-a) are considered the most important requirements for algal growth [137,146]. Salinity has been shown to affect physiology, growth, and community shifts in marine algae, especially in estuaries, where salinity differences are high [147–149] However, salinity has been rarely considered as a variable controlling algal settlement and growth in coral reefs [48,134,146]. Our data indicate that in the high salinity of the central Red Sea already small differences can influence algal biofilm assemblages. It is important to note though, that our results represent the best match of the variables investigated. For algal biofilm settlement and succession, grazing is usually another highly influential driver [48,150], and herbivory may therefore have had a contribution in our study area as well.

### Conclusions

The Red Sea is known as an oligotrophic, but sparsely studied region that maintains reefs of high coral cover at high temperature and salinity. Our analyses highlight spatio-temporal dynamics of physico-chemical and biotic variables in the central Red Sea. As such, our data provide a comparative foundation for future coral reef studies. *In situ* data show that temperature and dissolved oxygen concentrations on reefs in this region are similar to projected ‘future ocean’ conditions on reefs elsewhere. Therefore, reefs in the central Red Sea provide an opportunity to study coral reef functioning under environmental change.

## Supporting Information

S1 Table Details on logging, data collection, and sampling design. (XLSX)

S2 Table Overview of sequence abundance counts per OTU and sample, OTU taxonomic classification, and OTU 16S rRNA reference sequence for bacterial communities of seawater and biofilms. (XLSX)

S3 Table Summary statistics for sequence and OTU-based alpha-diversity measures of bacterial communities of seawater and biofilm. (XLSX)

S4 Table Differentially abundant bacterial OTUs and algal groups of biofilms over reefs and seasons. (XLSX)
